# Evaluation of Transport Media and Specimen Transport Conditions for the Detection of SARS-CoV-2 by Use of Real-Time Reverse Transcription-PCR

**DOI:** 10.1128/JCM.00708-20

**Published:** 2020-07-23

**Authors:** Amy A. Rogers, Russell E. Baumann, Gwynngelle A. Borillo, Ron M. Kagan, Hollis J. Batterman, Marzena M. Galdzicka, Elizabeth M. Marlowe

**Affiliations:** a Quest Diagnostics Infectious Disease, San Juan Capistrano, California, USA; b Quest Diagnostics, Marlborough, Massachusetts, USA; Boston Children's Hospital

**Keywords:** RNA stability, SARS-CoV-2, transport conditions, transport media

## Abstract

The global coronavirus (CoV) disease 2019 (COVID-19) pandemic has resulted in a worldwide shortage of viral transport media and raised questions about specimen stability. The objective of this study was to determine the stability of severe acute respiratory syndrome CoV 2 (SARS-CoV-2) RNA in specimen transport media under various storage conditions. Transport media tested included UTM, UTM-RT, ESwab, M4, and saline (0.9% NaCl). Specimen types tested included nasopharyngeal/oropharyngeal swabs in the above-named transport media, bronchoalveolar lavage (BAL) fluid, and sputum. A high-titer SARS-CoV-2 remnant patient specimen was spiked into pooled SARS-CoV-2 RNA-negative specimen remnants for the various medium types. Aliquots of samples were stored at 18°C to 26°C, 2°C to 8°C, and −10°C to −30°C and then tested at time points up to 14 days. Specimens consistently yielded amplifiable RNA with mean cycle threshold differences of <3 over the various conditions assayed, thus supporting the use and transport of alternative collection media and specimen types under a variety of temperature storage conditions.

## Abstract (precis)

The global coronavirus (CoV) disease 2019 (COVID-19) pandemic has resulted in a worldwide shortage of viral transport media and raised questions about specimen stability. The objective of this study was to determine the stability of severe acute respiratory syndrome CoV 2 (SARS-CoV-2) RNA in specimen transport media under various storage conditions. Transport media tested included UTM, UTM-RT, ESwab, M4, and saline (0.9% NaCl). Specimen types tested included nasopharyngeal/oropharyngeal swabs in the above-named transport media, bronchoalveolar lavage (BAL) fluid, and sputum.

## INTRODUCTION

On 31 December 2019, an outbreak of respiratory disease caused by a novel coronavirus (CoV) first detected in Wuhan City, Hubei Province, China, was initially reported to the World Health Organization (WHO) and has continued to expand globally (https://www.fda.gov/emergency-preparedness-and-response/mcm-legal-regulatory-and-policy-framework/emergency-use-authorization#covidinvitrodev, accessed 8 April 2020; https://www.cdc.gov/coronavirus/2019-nCoV/summary.html). On 30 January 2020, the United States reported the first confirmed instance of person-to-person spread of SARS-CoV-2 to an individual who had had close contact with a known case (https://www.cdc.gov/coronavirus/2019-nCoV/summary.html). On 11 March 2020, the WHO declared the 2019 CoV disease (COVID-19) a pandemic (https://www.who.int/dg/speeches/detail/who-director-general-s-opening-remarks-at-the-media-briefing-on-covid-19---11-march-2020, accessed 28 April 2020).

Coronaviruses are a large family of viruses that are common in many different species, including camels, cattle, cats, and bats (https://www.cdc.gov/coronavirus/2019-nCoV/summary.html). Globally, there are now five common human coronaviruses in circulation: 229E, NL63, OC43, HKU1, and SARS-CoV-2. Infection and spread of animal coronaviruses into people are rare. Cases of zoonotic transmission of animal coronaviruses, including Middle East respiratory syndrome CoV (MERS-CoV), SARS-CoV-1, and SARS-CoV-2 (previously known as 2019 novel coronavirus [2019-nCoV]), have been described (https://www.cdc.gov/coronavirus/2019-nCoV/summary.html). Since the first reported cases of SARS-CoV-2 in December 2019, more than 2.6 million cases have been reported globally as of 22 April 2020, according to the COVID-19 dashboard maintained by the Center for Systems Science and Engineering at John Hopkins University (https://www.arcgis.com/apps/opsdashboard/index.html#/bda7594740fd40299423467b48e9ecf6).

The COVID-19 pandemic has resulted in an unprecedented worldwide demand for laboratory testing. The huge increase in testing has put pressure on the laboratory supply chain and resulted in shortages of viral transport media. The Food and Drug Administration (FDA) has recommended the use of alternative viral transport media, such as liquid Amies, saline, and phosphate-buffered saline (PBS). Further recommendations suggest that specimens can be stored for up to 72 h at 4°C or frozen at no more than −70°C for longer storage (https://www.fda.gov/medical-devices/emergency-situations-medical-devices/faqs-diagnostic-testing-sars-cov-2; accessed 2 April 2020). The objective of this study was to evaluate the detection of SARS-CoV-2 RNA by real-time reverse transcription-PCR (rRT-PCR), using pooled remnant respiratory specimens placed into different transport media and held under common specimen transport conditions.

## MATERIALS AND METHODS

### Clinical specimens.

Transport media tested at Quest Diagnostics Infectious Disease (QDID; San Juan Capistrano, CA) included UTM (Copan, Brescia, Italy), UTM-RT (Copan, Brescia, Italy), ESwab (Copan, Brescia, Italy), M4 medium (Thermo Fisher Scientific, Waltham, MA), and normal saline [0.9% NaCl]. Specimen types tested included nasopharyngeal/oropharyngeal (NP/OP) swabs in the various transport media, bronchoalveolar lavage (BAL) fluid, and sputum. Prior to spiking, remnant sputum had been processed in 1× PBS. A known high-titer SARS-CoV-2-positive specimen was utilized to spike SARS-CoV-2 RNA-negative specimen remnants for the various media. The RNA viral load was estimated based on cycle threshold (*C_T_*) values to be approximately 1,500 copies/ml.

### Study design.

Samples were aliquoted and stored for up to 14 days and tested at multiple time points and storage temperatures (18°C to 26°C, 2°C to 8°C, and –10°C to –30°C). Five aliquots were assayed under each condition. Molecular analysis utilizing the QDID SARS-CoV-2 RNA qualitative real-time RT-PCR emergency use authorization (EUA) assay was performed according to the package instructions for use (https://www.fda.gov/emergency-preparedness-and-response/mcm-legal-regulatory-and-policy-framework/emergency-use-authorization#covidinvitrode, accessed 8 April 2020). A positive result for SARS-CoV-2 RNA was defined as a *C_T_* value of <40 for both the N1 and N3 detectors. Samples were tested before storage (time zero) to obtain an initial *C_T_* result. Samples were deemed stable if the mean *C_T_* values did not increase by more than 3 amplification cycles of the mean initial *C_T_* value.

### Additional saline studies.

Storage studies using normal saline [0.9% NaCl] were also performed at Quest Diagnostics, Marlborough, MA, using the Quest Diagnostics and the Roche Diagnostics cobas SARS-CoV-2 EUA tests. A high-titer SARS-CoV-2-positive patient specimen with a *C_T_* of 18 as established by the Roche cobas system was diluted 1:1,000 to obtain a calculated *C_T_* value of 28. For each time point, samples were tested in triplicate at this concentration. A further 1:10 dilution of this material (calculated *C_T_* = 31) was also tested in duplicate at each time point.

### Statistical analysis.

Statistical analysis was performed using Analyze-it for Microsoft Excel, version 5.40.2.

## RESULTS

SARS-CoV-2 RNA was consistently detected in all transport media, specimen types, and storage conditions tested; mean *C_T_* values obtained for SARS-CoV-2 for the various study-defined transport media and storage temperatures are shown in Tables 1 to 3. Differences in average viral RNA *C_T_* values were similar across all media and temperature storage conditions assayed (Tables 1 to 3). Mean *C_T_* differences between day 0 and day 7 for all media tested at room temperature were 0.6 ± 0.7 *C_T_*. Refrigerated and frozen samples exhibited mean *C_T_* differences of 0.5 ± 0.7 and 0.7 ± 1.1 *C_T_*, respectively. For frozen ESwab samples, one of five replicates at day 5 did not yield detectable RNA for the N3 target (Table 3). Detection of only one of two targets in the assay is considered an inconclusive result. For saline and ESwab transport media, there was a shift of >2 *C_T_*s in the average *C_T_* between day 0 and day 7 and/or day 14 (Tables 1 to 4). These changes would not have altered the interpretation of positive results.

**TABLE 1:** Stability of room temperature SARS-CoV-2 RNA detected by the Quest EUA rRT-PCR^*a*^

Medium or sample type	Detector^*b*^	Mean *C_T_* (SD) of 5 specimens at room temp (18 to 26°C) on day:				
		0	2	3	5	7
UTM	N1	31.8 (0.2)	31.6 (0.4)	31.6 (0.3)	31.6 (0.4)	31.5 (0.8)
	N3	31.3 (0.4)	30.8 (0.2)	30.8 (0.5)	30.8 (0.4)	30.7 (0.7)
UTM-RT	N1	31.8 (0.3)	31.6 (0.3)	31.3 (0.2)	31.9 (0.4)	33.1 (0.4)
	N3	31.2 (0.4)	30.8 (0.3)	31.0 (0.2)	31.1 (0.3)	32.2 (0.4)
ESwab	N1	31.7 (0.4)	32.0 (0.4)	31.9 (0.5)	31.9 (0.4)	32.6 (1.0)
	N3	31.3 (0.3)	31.1 (0.2)	30.9 (0.3)	31.0 (0.3)	31.6 (0.7)
M4	N1	31.8 (0.2)	32.1 (0.4)	31.5 (0.2)	32.4 (0.5)	32.6 (0.6)
	N3	31.3 (0.4)	31.2 (0.4)	31.0 (0.1)	31.2 (0.2)	31.6 (0.4)
Saline	N1	29.2 (0.7)	29.9 (0.3)	30.3 (0.1)	30.7 (0.3)	31.1 (0.3)
	N3	28.4 (0.6)	29.1 (0.2)	29.5 (0.1)	29.9 (0.5)	30.0 (0.2)
BAL fluid	N1	31.8 (0.2)	31.5 (0.2)	32.2 (0.5)	31.4 (0.3)	32.4 (0.4)
	N3	31.1 (0.2)	30.8 (0.2)	31.2 (0.4)	30.6 (0.3)	31.2 (0.4)
Sputum	N1	31.4 (0.5)	31.8 (0.6)	32.1 (0.4)	31.8 (0.4)	32.1 (0.3)
	N3	30.8 (0.6)	31.1 (0.7)	31.1 (0.5)	30.8 (0.3)	31.1 (0.3)

a Testing was performed at QDID. Five replicates were assayed by rRT-PCR under the indicated conditions. UTM, medium for clinical specimens containing viruses, chlamydiae, mycoplasma, or ureaplasma; UTM-RT, universal transport medium for viruses, mycoplasma, and ureaplasma.

b N1 and N3 TaqMan probes were as described in the Quest Diagnostics EUA package insert.

**TABLE 2:** Stability of refrigerated SARS-CoV-2 RNA detected by the Quest EUA rRT-PCR^*a*^

Medium or sample type	Detector^*b*^	Mean *C_T_* (SD) of 5 refrigerated (2 to 8°C) specimens on day:					
		0	3	5	7	10	14
UTM	N1	31.8 (0.2)	31.8 (0.2)	31.8 (0.4)	31.9 (0.6)	32.2 (0.4)	32.1 (0.3)
	N3	31.3 (0.4)	31.3 (0.4)	31.1 (0.5)	31.0 (0.5)	31.6 (0.4)	31.4 (0.4)
UTM-RT	N1	31.8 (0.3)	31.4 (0.3)	32.1 (0.1)	31.1 (1.8)	32.5 (0.2)	32.1 (0.3)
	N3	31.2 (0.4)	30.8 (0.4)	31.3 (0.2)	30.4 (1.7)	31.6 (0.1)	31.4 (0.3)
ESwab	N1	31.7 (0.4)	31.9 (0.3)	31.7 (0.2)	31.8 (0.3)	31.7 (0.4)	31.8 (0.4)
	N3	31.3 (0.3)	31.0 (0.4)	30.7 (0.2)	30.7 (0.3)	30.8 (0.4)	31.2 (0.4)
M4	N1	31.8 (0.2)	31.7 (0.3)	32.0 (0.4)	31.5 (1.5)	31.8 (0.5)	31.9 (0.2)
	N3	31.3 (0.4)	31.0 (0.3)	31.1 (0.3)	30.7 (1.2)	31.3 (0.6)	31.3 (0.3)
Saline	N1	29.2 (0.7)	30.1 (0.2)	30.0 (0.4)	30.1 (1.7)	30.4 (1.2)	31.3 (0.4)^*c*^
	N3	28.4 (0.6)	28.9 (0.2)	29.0 (0.5)	28.9 (1.4)	29.4 (1.0)	30.5 (0.4)^*c*^
BAL fluid	N1	31.8 (0.2)	32.2 (0.6)	31.0 (1.4)	32.5 (0.1)	32.1 (0.6)	32.4 (0.7)
	N3	31.8 (0.2)	32.2 (0.6)	31.0 (1.4)	32.5 (0.1)	32.1 (0.6)	31.5 (0.5)
Sputum	N1	31.4 (0.5)	31.4 (0.5)	31.8 (0.6)	32.1 (0.5)	32.1 (0.4)	32.2 (0.7)
	N3	30.8 (0.6)	30.7 (0.9)	31.0 (0.4)	31.0 (0.4)	31.8 (0.6)	31.6 (0.8)

a Testing was performed at QDID. Five replicates were assayed by rRT-PCR under the indicated conditions.

b N1 and N3 TaqMan probes were as described in the Quest Diagnostics EUA package insert.

c Increase of >2 *C_T_*s from day 0.

**TABLE 3:** Stability of frozen SARS-CoV-2 RNA detected by the Quest EUA rRT-PCR^*a*^

Medium or sample type	Detector^*b*^	Mean *C_T_* (SD) of 5 frozen (−10 to −30°C) specimens on day:				
		0	3	5	7	14
UTM	N1	31.8 (0.2)	31.9 (0.4)	31.5 (0.3)	32.0 (0.4)	32.0 (0.4)
	N3	31.3 (0.4)	31.1 (0.3)	30.6 (0.4)	31.1 (0.3)	31.2 (0.4)
UTM-RT	N1	31.8 (0.3)	31.7 (0.4)	31.9 (0.4)	32.3 (0.2)	31.7 (0.4)
	N3	31.2 (0.4)	31.2 (0.3)	31.0 (0.4)	31.4 (0.4)	31.0 (0.2)
ESwab	N1	31.7 (0.4)	34.3 (1.1)	34.8 (0.4)	31.8 (0.3)	33.5 (0.6)
	N3	31.3 (0.3)	33.7 (0.9)	34.5 (0.4)^*d*^	31.7 (0.4)	33.8 (0.6)^*c*^
M4	N1	31.8 (0.2)	32.1 (0.7)	32.0 (0.5)	31.0 (3.0)	31.5 (0.2)
	N3	31.3 (0.4)	31.6 (0.7)	31.1 (0.5)	30.1 (3.0)	31.0 (0.3)
Saline	N1	29.2 (0.7)	30.6 (0.5)	31.4 (0.3)	31.7 (0.4)^*c*^	31.6 (0.9)^*c*^
	N3	28.4 (0.6)	29.6 (0.4)	30.3 (0.3)	30.6 (0.4)^*c*^	30.7 (0.9)^*c*^
BAL fluid	N1	31.8 (0.2)	32.4 (0.6)	32.2 (0.4)	32.5 (0.3)	32.5 (0.1)
	N3	31.1 (0.2)	31.3 (0.4)	31.3 (0.3)	31.8 (0.3)	31.6 (0.2)
Sputum	N1	31.4 (0.5)	31.8 (1.0)	31.6 (0.3)	31.8 (0.5)	31.8 (0.3)
	N3	30.8 (0.6)	30.9 (1.0)	30.7 (0.1)	30.8 (0.5)	31.0 (0.4)

a Testing was performed at QDID. Five replicates were assayed by rRT-PCR under the indicated conditions.

b N1 and N3 TaqMan probes were as described in the Quest Diagnostics EUA package insert.

c Increase of >2 *C_T_*s from day 0.

d One of five replicates at day 5 did not yield detectable RNA for the N3 target.

**TABLE 4:** Additional Stability of SARS-CoV-2 RNA in Saline detected by the Quest EUA and Roche cobas EUA^*a*^

Specimen condition	Platform	Detector^*b*^	Mean *C_T_* (SD) of 3 specimens on day:						
			0	1	2	3	5	7	14
Room temp (18 to 26°C)	Quest	N1	26.2 (0.1)	26.3 (0.0)	26.1 (0.5)	26.8 (0.1)	27.2 (0.1)	26.4 (0.1)	26.8 (0.4)
		N3	30.2 (0.1)	30.3 (0.1)	30.0 (0.4)	30.7 (0.1)	31.5 (0.1)	29.3 (0.1)	29.3 (0.3)
	cobas	ORF1a/b	27.9 (0.3)	28.4 (0.2)	28.5 (0.1)	29.1 (0.1)	29.4 (0.2)	29.5 (0.0)	30.0 (0.2)^*c*^
		E gene	28.1 (0.3)	28.6 (0.2)	28.7 (0.1)	29.3 (0.2)	29.7 (0.2)	29.9 (0.2)	30.4 (0.2)^*c*^

Refrigerated (2 to 8°C)	Quest	N1	26.2 (0.1)	26.4 (0.1)	26.0 (0.2)	26.5 (0.0)	27.0 (0.1)	26.3 (0.2)	26.5 (0.2)
		N3	30.2 (0.1)	30.2 (0.1)	29.8 (0.2)	30.6 (0.0)	31.3 (0.1)	29.0 (0.2)	29.1 (0.1)
	cobas	ORF1a/b	27.9 (0.3)	28.5 (0.1)	28.5 (0.1)	28.8 (0.1)	29.2 (0.1)	29.3 (0.1)	29.9 (0.1)
		E gene	28.1 (0.3)	28.8 (0.1)	28.7 (0.1)	29.1 (0.1)	29.5 (0.1)	29.5 (0.2)	30.2 (0.2)^*c*^

Frozen (−60 to −90°C)	Quest	N1	26.2 (0.1)	26.3 (0.0)	26.1 (0.5)	26.8 (0.1)	27.2 (0.1)	26.4 (0.1)	26.8 (0.4)
		N3	30.2 (0.1)	30.3 (0.1)	30.0 (0.4)	30.7 (0.1)	31.5 (0.1)	29.3 (0.1)	29.3 (0.3)
	cobas	ORF1a/b	27.9 (0.3)	28.4 (0.1)	28.2 (0.2)	28.7 (0.2)	28.5 (0.2)	28.3 (0.1)	28.4 (0.1)
		E gene	28.1 (0.3)	28.7 (0.2)	28.4 (0.3)	28.7 (0.1)	28.8 (0.1)	28.6 (0.1)	28.7 (0.0)

a Testing was performed at Quest Diagnostics, Marlborough, MA. Three replicates were assayed by rRT-PCR at each of the indicated conditions.

b N1 and N3 TaqMan probes were as described in the Quest Diagnostics EUA package insert, and ORF1a and ORF1b (ORF1a/b) and E gene probes were as described in the Roche Diagnostics EUA package insert.

c Increase of >2 *C_T_*s from day 0.

A further stability study performed at a second site assessed the stability of SARS-CoV-2 RNA in saline for up to 14 days using both the Quest and the Roche cobas EUA assays. As shown in Table 4, SARS-CoV-2 RNA remained detectable for 14 days on both platforms. For the samples tested by the Quest and the cobas EUA assays on frozen saline, we observed minimal variation (<1 *C_T_* on average) in the mean *C_T_* values over 14 days. However, at room temperature and under refrigerated storage conditions, we noted a >2 *C_T_* increase over 14 days (Table 4). The increase in the *C_T_* values was linear over 14 days, with slopes of 0.14 and 0.15 *C_T_*s per day for the two targets (*R*^2^, 0.79 and 0.78) at room temperature and 0.13 *C_T_* per day (*R*^2^, 0.83 and 0.81) with refrigerated storage. A further 1:10 dilution of the SARS-CoV-2 RNA (calculated *C_T_* = 31) was also tested in duplicate at each time point, and comparable results and trends were observed (data not shown).

## DISCUSSION

Limited-stability studies are available in the literature for SARS-CoV-2 RNA. A study of SARS-CoV-2 in aerosols and on surfaces demonstrated that culturable SARS-CoV-2 was detectable in aerosols for up to 3 h, up to 4 h on copper, up to 24 h on cardboard, and up to 2 to 3 days on plastic and stainless steel (1). Given the persistence of SARS-CoV-2 in the environment, it is not surprising that RNA can be reliably amplified from viral transport media after relatively long storage times, even at room temperature.

Prior stability studies of SARS-CoV-2 RNA in various transport media and conditions are also limited. The results reported here are consistent with the findings of Druce et al. (2), who examined the stability of four common viruses with different physicochemical properties in several swab and transport media and storage combinations. The authors demonstrated that influenza virus, enterovirus, herpes simplex virus, and adenovirus were detected by PCR at 22°C and after refrigeration at 4°C for up to 7 days.

Stability using various viral transport media performed with in-house laboratory-developed tests for influenza and rubeola viruses (both enveloped RNA viruses) are also consistent with viral RNA detection at 18 to 26°C after 7 days, 2 to 8°C after 14 days, and −10 to –30°C after 30 days (data not shown). Rodino et al. (3) demonstrated reliable detection of SARS-CoV-2 RNA in swabs stored in minimal essential medium (MEM), PBS, saline, and viral transport medium (VTM) after 7 days at 2 to 8°C and frozen at –20°C using an in-house EUA assay as well as the Roche cobas EUA assay.

Qualitative detection of SARS-CoV-2 RNA was unchanged over the various combinations of transport media and conditions tested at two study sites, regardless of molecular platform utilized. While stability in saline stored at room temperature and under refrigerated conditions exhibited a linear trend with increasing *C_T_* values over time, these trends did not impact the qualitative interpretation of positive results. Additionally, specimen stability was assessed in this study for a significantly longer duration than would be deemed acceptable for routine clinical testing, further reducing the probability of clinical impact. The greatest theoretical impact of this linear trend is for specimens with low titers of virus. In our experience with clinical specimens, however, the majority of positive specimens tested exhibited low *C_T_* values (<30), which correlate with specimens with higher titers of virus. Viral titers may be impacted by the clinical course at the time of sample acquisition and by the quality of the specimen collection. These data provide additional supporting evidence for the use of alternative viral transport media and temperature storage conditions for the detection SARS-CoV-2 RNA using sensitive rRT-PCR assays.
